# The prevalence of anemia and the factors associated with its severity among children aged 6–59 months in Ghana: A multi-level ordinal logistic regression

**DOI:** 10.1371/journal.pone.0315232

**Published:** 2024-12-23

**Authors:** Yordanos Sisay Asgedom, Aklilu Habte, Beshada Zerfu Woldegeorgis, Mengistu Meske Koyira, Beimnet Desalegn Kedida, Bezawit Melak Fente, Amanuel Yosef Gebrekidan, Gizachew Ambaw Kassie

**Affiliations:** 1 Department of Epidemiology, College of Health Sciences and Medicine, Wolaita Sodo University, Soddo, Ethiopia; 2 School of Public Health, College of Medicine and Health Sciences, Wachemo University, Hosanna, Ethiopia; 3 School of Medicine, College of Health Sciences and Medicine, Wolaita Sodo University, Soddo, Ethiopia; 4 School of Public Health, College of Health Sciences and Medicine, Wolaita Sodo University, Soddo, Ethiopia; 5 Department of General Midwifery, School of Midwifery, College of Medicine & Health Sciences, University of Gondar, Gondar, Ethiopia; Kintampo Health Research Centre, GHANA

## Abstract

**Introduction:**

Anemia is a significant health problem that has a profound impact on young children under the age of five. It can result in severe consequences, such as stunted growth, impaired cognitive and motor development, increased illness, and even death. In Ghana, anemia is the leading cause of child mortality, yet there is a lack of information available on the prevalence of anemia and the factors associated with its severity in children under five in the country. To fill this gap, this study was conducted to investigate the prevalence and determinants of anemia severity among children aged 6–59 months in Ghana.

**Methods:**

This study used data from the 2022 Ghana Demographic and Health Survey (GDHS) and included a weighted total sample of 3585 children aged 6–59 months. Given the hierarchical nature of the DHS data and the ordinal nature of anemia, a multilevel ordinal logistic regression model was employed. The Brant test was used to determine whether the proportional odds assumption was met (P ≥0.05). Deviance was used for model comparison. For the multivariable analysis, variables with a p-value ≤0.2 in the bi-variable analysis were considered. The Adjusted Odds Ratio (AOR) with a 95% Confidence Interval (CI) was reported as a factor associated with anemia severity in the multivariable multilevel proportional odds model.

**Results:**

A study conducted in Ghanaian children aged 6–59 months found that 49.1% of them had anemia (95% CI: 47.4%-50.7%). The results indicated that 27.6% of patients had mild anemia, while 21.4% had moderate to severe anemia. Factors that were significantly associated with higher odds of childhood anemia included being 6–23 months old, male, having a maternal age of 15–24 or 25–34 years, belonging to poorer or wealthier households, having a higher birth order, being stunted, having maternal anemia, no media exposure, and living in the Northern, Upper East, or Upper West regions.

**Conclusion:**

Anemia among children aged 6–59 months in Ghana is a major public health concern. It is recommended to improve access to the media, address maternal anemia through targeted interventions, and strengthen the wealth status of families. Furthermore, preconception care for mothers during pregnancy should be supported to reduce anemia in the long-term. Additionally, the early detection and management of stunted children should be strengthened to decrease childhood anemia.

## Introduction

Anemia is a condition in which the number of red blood cells or the concentration of haemoglobin inside them is lower than normal [1]. According to the World Health Organization (WHO) anemia is defined as a blood haemoglobin level of <11 g/dl in children aged 6–59 months. This is further categorized as severe, moderate, and mild anemia with different cut-off points [2]. Infections such as HIV, tuberculosis, malaria, parasitic infections, and nutrient deficiencies (folate, iron, vitamin B12 and A) are the most common causes of anemia in children [1].

Anemia is a serious global public health problem that particularly affects young children and pregnant women [3, 4]. Anemia affects 269 million 6–59 months old children [1]. Based on the 2019 WHO report, 40% of 6–59 months old children across the globe were anemic [1]. Africa and Southeast Asia are the most affected regions, with an estimated 103 million children affected by anemia in Africa and 83 million children affected in Southeast Asia [1]. The prevalence notably high due to factors include malnutrition, limited healthcare infrastructure, and a high prevalence of infectious diseases such as malaria and HIV/AIDS, which increase the risk of anemia [5, 6]. Anemia is most prevalent in central and western subregions of Africa [7].

According to the recent 2022 Ghana Demographic and Health Survey, the prevalence of anemia among 6–59 months old children in Ghana is 49%, indicating that anemia is still a major public health problem in the country [8]. Anemia has severe consequences on the health of children. It exerts a profoundly negative effect on children’s health, leading to impaired cognitive development, which manifests as diminished academic performance and learning difficulties. Additionally, it is associated with developmental delays, challenges in concentration, weakened immune function, higher mortality rates, increased fatigue, and a greater vulnerability to infections [9].

The leading causes of anemia in sub-Saharan African countries like Ghana are nutritional deficiencies and infectious diseases, although the causes of anemia are multifactorial and vary according to socioeconomic factors [10–12]. Anemia among children is associated with child age, maternal age, birth order, residence, deworming, place of delivery, household wealth status, maternal anemia, maternal educational status, malaria, hookworm, childhood nutritional status [13–17]. Which disproportionately affects poorer households, socially marginalized populations and those with no formal education background [2 socio]. Previous evidence also indicated that anemia was concentrated among poorer households [18, 19]. Several demographic characteristics are linked to childhood anemia, including child age, maternal age, residence, household wealth status, and maternal educational status [13–17].

Despite significant improvements in the socioeconomic and health conditions of many communities around the world, Ghana continues to struggle with a large number of under-five deaths [20]. In order to meet the objective of decreasing child mortality as part of the Sustainable Development Goals (SDGs) for the year 2030, it is necessary to determine the severity of anemia in children aged 6–59 months and identifying the factors that contribute to it is crucial for creating effective prevention and treatment strategies. However, there have been limited studies on the factors that determine the severity of anemia (including none, mild, moderate-severe anemia) among children in Ghana [21]. Furthermore, to obtain a precise and reliable estimate without any loss of information, we utilized a multilevel ordinal logistic regression. Thus, this study aimed to investigate the various levels of anemia and the individual, household, and community-level factors that contribute to it by utilizing the 2022 Ghana Demographic and Health Survey (GDHS) report. The results of this study could assist policymakers in prioritizing and investing in cost-effective interventions and policies that can help reduce the prevalence of anemia in children.

## Methods

### Data source and sampling procedure

**Fig 1:**
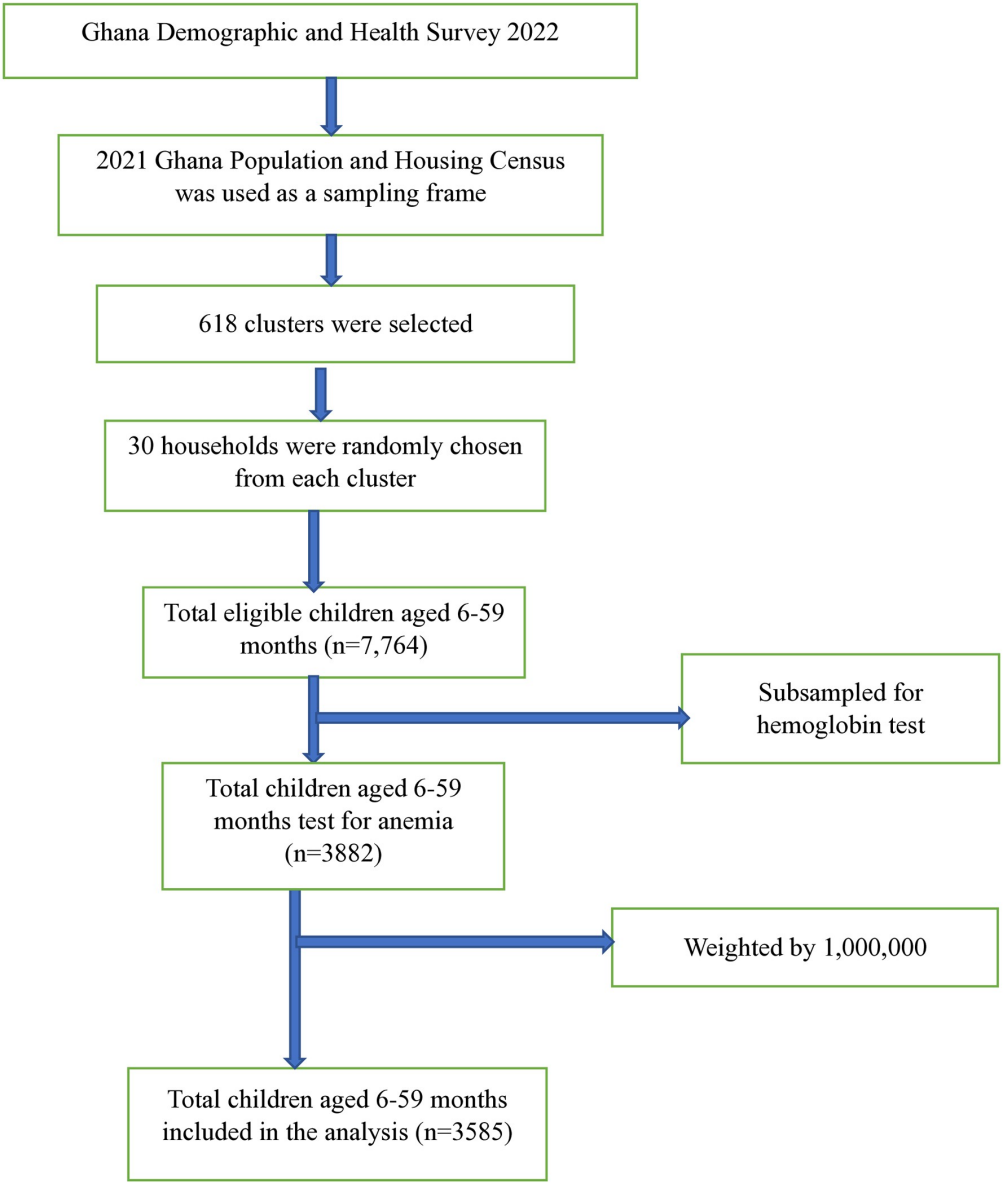
Sampling procedure and sampling technique anemia status and its determinants among children aged 6–59 months using 2022 GDHS.

We conducted cross-sectional analyses to assess the the prevalence of anemia and the factors associated with its severity among children aged 6–59 months in Ghana. Demographic and Health Surveys (DHS) are nationally representative household surveys designed to collect data on wide range of health and population indicators including socio-demographic, maternal and child health nutrition. The DHS follows a multistage stratified sampling design with the primary administrative units as urban and rural strata. At first stage, a random selection of enumeration areas (EAs) are randomly selected within each stratum followed by selection of fixed number of households from each EA. DHS surveys consist of Standard DHS Surveys, which have large sample sizes (5,000 to 30,000 households) conducted every five years for time comparisons [22]. We used the most recent survey data of Ghana Demographic and Health Survey (GDHS) and the study used the Kids Record (KR) file with final number of analytic participants totaling 3,585 children aged 6–59 months (Fig 1).

### Variables of the study and measurements

#### Dependent variable

The outcome of interest in our study was anemia, which was classified as severe, moderate, mild, or none based on the WHO’s Hb cutoff points (Mild: 10.0–10.9, Moderate: 7.0–9.9 and severe <7.0) for diagnosis [2]. Blood samples were collected from children aged 6–59 months after obtaining consent from their parents or guardians. A drop of blood was obtained via a finger prick, or a heel prick for children aged 6–11 months, and placed in a microcuvette. Hemoglobin levels were analyzed on-site using a portable, battery-operated HemoCue^®^ 201+ device. In DHS the altitude adjustment was made because prolonged exposure to hypoxia and decreased partial pressure of oxygen at high altitudes enhance erythropoiesis and raise Hb levels [23]. It was evaluated by measuring blood hemoglobin concentration, adjusted for altitude. At elevations above 1000 meters, hemoglobin levels rise as an adaptive response to the lower oxygen pressure and decreased blood oxygen saturation. This compensatory rise in red blood cell production helps ensure adequate oxygen delivery to tissues [8, 23].


$$\text{Hb}=-0.32\times \left(\text{altitude}\;\text{in}\;\text{meters}\times .0033\right)+0.22{\left(\text{altitude}\;\text{in}\;\text{meters}\times .0033\right)}^{2}$$

#### Independent variables

Because of the hierarchical nature of the DHS data, the independent variables in the study were obtained from two sources: individual and community-level variables. Household-related characteristics, child-related characteristics, and maternal-related characteristics were considered as individual-level factors. Household-related factors included the availability of an improved water source, type of toilet, number of household members, media exposure, and household wealth status. Child-related factors included the sex of the child, age of the child, type of birth, birth order, deworming, vitamin A supplementation in the last six months, diarrhea in the last two weeks, fever in the last two weeks, cough in the last two weeks, and underweight status (defined as a weight-for-age Z-score (WAZ) < -2SD). Maternal-related factors included maternal age, employment status, maternal education, antenatal care visit status (ANC), maternal anemia, iron supplementation during pregnancy, place of delivery, wanted birth, history of cigarette smoking, and maternal body mass index (BMI) and maternal anemia autonomy in decision making. Residence and regions were considered as level-two variables.

Underweight was defined as children with a weight-for-age Z-score (WAZ) < -2SD, wasting was defined as children with a weight-for-height Z-score (WHZ) < -2SD, and stunting was defined as children with a height-for-age Z-score (HAZ) < -2SD. Maternal anemia was categorized into anemic (<7 g/dl—10.9 g/dl) and not anemic (>11 g/dl) for pregnant women, and for non-pregnant women, it was categorized as not anemic (>12 g/dl) and (87 g/dl—11.9 g/dl), respectively.

### Operational definition

#### Media exposure

The variable for media exposure is based on three activities: listening to the radio, watching television, and reading newspapers or magazines. Women who participated in any of these activities at least once a week were classified as having media exposure (coded as "Yes"), while those who did not engage in these activities were classified as not having media exposure (coded as "No") [24].

#### Autonomy in decision-making

Was assessed by compiling and categorising replies to three questions about who takes the final decision for the family on big property purchases, visits to relatives, and health care. The response categories were (i) woman alone, (ii) woman and husband/partner, (iii) husband/partner alone, (iv) someone else, and (v) others. For each question, responses (i) or (ii) got a score of 1, indicating good decision-making capacity, whereas the remaining responses received a score of 0, indicating weak decision-making capacity. Each of the three components’ responses were summed together to yield an overall score ranging from 0 to 3. Finally, a composite score was divided into two distinct groups: low and high for "0 to 2" and "3" scores [25, 26].

### Data management and analysis

The data before conducting any statistical analysis was adjusted for representativeness and sampling design using sampling weights, primary sampling units, and strata. This was done to acquire dependable statistical estimates. STATA (StataCorp, USA) version 17 statistical software was utilized for data management and analysis. In this study, the outcome variable was polychotomous and exhibited an ordinal nature, necessitating the use of an ordinal logistic regression model to evaluate the predictors of anemia (not anemic, mild, moderate-severe anemia). In bivariable analysis the variables with a p-value equal to or less than 0.2 were selected for the multivariable multilevel proportional odds model. An ordinal logistic regression model was utilized because the fundamental assumption is proportional odds. If this assumption is met, Proportional Odds (PO) model can be used; otherwise, a partial proportional model should be used. To determine the appropriate ordinal model for the data, we evaluated the Proportional Odds (PO) assumptions, which state that the effects of all independent variables are constant across categories of the outcome variable.

Multilevel ordinal logistic regression models ordinal outcomes while accounting for hierarchical data structures.

The equation for a multilevel ordinal logistic is [27].


$$log\left[\frac{Pijc}{1-Pijc}\right]=\gamma_{c}-\left[x_{ij\beta +}v_{i}\right]$$

In this equation, the terms are:

*γ*_*c*_: The model thresholds, which are similar to intercepts and indicate the number of responses in each category

*x*_*ij*_: The covariates

*β*: The regression slopes, or the effects of the covariates

*v*_*i*_: The cluster effect, which represents the effect of the cluster on the subject’s outcome

After applying the proportional odds model, Brant’s test was used to evaluate the proportional odds assumption, which examines the null hypothesis that there is no variation in the impact of the independent variables across different anemia levels. The test results demonstrated that the proportional odds assumption was met (p>0.05), allowing us to employ the proportional odds model to assess the factors influencing anemia levels and the independent variables.

The Brant test was used to verify the parallel-line assumption, which asserts that there is no difference in the impact of independent variables across different levels of anemia. The null hypothesis for the proportional odds assumption posits no disparity in the effects of independent variables.

Likelihood Ratio(LR) test, Intra-class Correlation Coefficient (ICC) were computed to measure the variation across clusters. The degree of heterogeneity of anemia between clusters was quantified by ICC [28].

$$\text{ICC}=\sigma^{2}/(\sigma^{2}+\pi^{2}/3)$$

Where: the standard logit distribution has a variance of *π*^2^/3, $\sigma_{\mu }^{2}$ indicates the cluster variance.

Four models were constructed for the multilevel logistic regression analysis. The first model was a null model without any explanatory variables to assess the impact of cluster variation on anemia levels. The second model was adjusted for individual-level variables, the third model was adjusted for community-level variables, and the fourth model was fitted with both individual and community-level variables simultaneously. Model comparison was conducted based on deviance (-2Log-Likelihood Ratio (LLR)), as the models were nested, and the model with the lowest deviance was considered the best-fitted model for the data.

The variables with a p-value equal to or less than 0.2 were selected for the multivariable multilevel proportional odds model. In this model, the Adjusted Odds Ratio (AOR) along with the 95% Confidence Interval (CI) was presented to indicate the strength of association, and a p-value of less than 0.05 was set as the threshold for statistical significance in the final model.

### Ethical consideration

The study conducted was a secondary analysis of publicly available survey data obtained from the MEASURE DHS program. The **MEASURE DHS Program** is a global initiative that collects and analyzes data on population, health, and nutrition in developing countries. Funded by USAID, it provides key information on topics such as maternal and child health, family planning, and HIV, supporting evidence-based policymaking and health program development. Since no original data collection was involved, the research was exempted from obtaining ethical approval and participant consent. Permission to access and utilize the data was granted by [www.dhsprogram.com]. It is essential to mention that the datasets contained no personal information, such as names or household addresses, to safeguard the privacy of the individuals involved.

## Results

### Study participant’s descriptive characteristics

The DHS data has a nested structure, with children and mothers grouped within clusters. It is assumed that individuals within the same cluster share similar characteristics as those in other clusters. However, this violates the assumptions of independence of observations and equal variance between clusters in the ordinal logistic regression model. Therefore, it is crucial to account for the heterogeneity between clusters using an advanced model. To address this, a multilevel cumulative Logit model was employed.

Since the Brant test was satisfied, a multilevel proportional odds model was used to calculate a single Odds Ratio (OR) for an explanatory variable (severe-moderate vs mild/not anaemic, mild/moderate-severe vs. not anaemic). Anemia, which was classified as severe, moderate, mild, or none based on the WHO’s Hb cutoff points for diagnosis [2]. However, due to the small number of children with severe anemia (n = 44), we merged them with the moderate category for the purpose of analysis, and referred to them as ’moderate-to-severe’ anemia. Additionally, many studies have utilized this approach to stabilize the model they planned to use for analysis [29].

A total of 3,585 children aged 6–59 months were included in the study, out of which 2230 (62.3%) were aged ≥24 months and 1806 (50.3%) were male (Table 1). Approximately 1348 (37.6%) were from parity 2–3, 846 (23.6%) were from parity 4–5, and 3430 (95.6%) were single births. Almost three-fourths (74.5%) and 1561 (43.5%) of children received Vitamin A and drug supplementation for intestinal parasites in the last six months. Approximately 3103 (86.5%), 2836 (79.1%), and 2996 (83.6%) of children did not have diarrhoea, cough, or fever in the last two weeks. Approximately 3117 (87.1%) and 3354 (93.6%) of children were not underweight or wasted. Nearly one-fifth (17.4%) of children were stunted. Almost half (49.3%) of mothers were aged 25–34, and 1880 (52.4%) had attained secondary education. Most (56.2%) of mothers had a normal body mass index, and 2156 (60.1%) were not anaemic. Approximately 1706 (89.6%) attended ≥4 ANC visits. Approximately 3288 (91.7%) of mothers gave birth at a health facility, and nearly half (52.3%) of mothers were iron supplemented during their pregnancy. Regarding household wealth status, approximately 827 (23.0%) were the poorest, and about 2019 (56.3%) had ≤5 household members. The majority (84.1%) had access to an improved water source, and 2018 (57.1%) used improved toilet facilities. Most (78.7%) of mothers had media exposure, and 2123 (59.2%) of mothers had autonomy in decision-making (Table 1).

**Table 1:** Individual-level characteristics of the study participants in Ghana.

Variable	Weighted frequency	Percentage (%)
**Child age in months**		
6–23	1354	37.7
24–59	2230	62.2
**Sex of child**		
Male	1806	50.3
Female	1778	49.6
**Type of birth**		
Single	3430	95.6
Multiple	154	4.3
**Birth order**		
1	933	26.0
2–3	1348	37.6
4–5	846	23.6
≥6	455	12.7
**Vitamin A supplementation in the last six months**		
Yes	2670	74.5
No	914	25.5
**Drugs for intestinal parasites in the last six months**		
Yes	1561	43.5
No	2024	56.5
**Diarrhoea in the last two weeks**		
Yes	481	13.4
No	3103	86.5
**Cough in the last two weeks**		
Yes	746	20.8
No	2836	79.1
**Fever in the last two weeks**		
Yes	587	16.3
No	2996	83.6
**Underweight status**		
Normal	3117	87.1
Underweight	460	12.8
**Wasting**		
Normal	3354	93.6
Wasted	226	6.3
**Stunting status**		
Normal	2951	82.5
Stunted	623	17.4
**Maternal age**		
15–24	681	19.0
25–34	1766	49.3
35–49	1136	31.7
**Maternal educational status**		
No education	858	23.9
Primary	533	14.8
Secondary	1880	52.4
Higher	312	8.7
**Maternal anemia status**		
Not anaemic	2156	60.1
Anaemic	1426	39.8
**Body mass index of mothers**		
Normal	2180	56.2
Underweight	213	5.5
Overweight	957	24.7
Obese	525	13.5
**ANC visits during pregnancy (N = 1903)**		
<4	197	10.3
≥4	1706	89.6
**Place of delivery**		
Home	294	8.2
Health facility	3288	91.7
**Supplementation of iron during pregnancy**		
Yes	1876	52.3
No	1706	47.6
**History of cigar ate smoking**		
Yes	3548	98.9
No	37	1.0
**Number of household members**		
≤5	2019	56.3
>5	1563	43.6
**Household wealth status**		
Poorest	827	23.0
Poorer	720	20.1
Middle	675	18.8
Richer	694	19.3
Richest	665	18.5
**Availability of improved water source**		
Improved	3016	84.1
Not improved	518	15.8
**Type of toilet**		
Improved	2018	57.1
Unimproved	461	13.0
Open defecation	1054	29.8
**Media exposure (N = 3533)**		
Yes	2783	78.7
No	750	21.2
**Decision Autonomy**		
Low	830	23.1
Moderate	631	17.6
High	2123	59.2

Regarding the community-level characteristics of the study participants, 1819 (50.7%) were from rural areas, 655 (18.2%) were from the Ashanti region, 458 (12.7%) were from Greater Accra, and 80 (2.2%) were from Ahafo (Table 2).

**Table 2:** Community-level characteristics of the study participants in Ghana.

Variables	Weighted frequency	Percentages (%)
**Residence**		
Urban	1763	49.2
Rural	1819	50.7
**Regions**		
Western	230	6.4
Central	362	10.1
Greater accra	458	12.7
Volta	140	3.9
Eastern	267	7.4
Ashanti	655	18.2
Western North	84	2.3
Ahafo	80	2.2
Bono	115	3.2
Bono east	181	5.0
Oti	114	3.1
Northern	399	11.1
Savannah	105	2.9
North East	107	3.0
Upper East	169	4.7
Upper West	116	3.2

#### Prevalence of levels of anemia

The prevalence of anemia among 6–59 months old Ghanaian children in this study was 49.1% (95% CI: 47.4%, 50.7%), with 27.6% (95% CI: 26.2%, 29.1%) experiencing mild anemia and 21.4% (95% CI: 20.1%, 22.7%) experiencing moderate-severe anemia.

### Random effect analysis results

#### Null model

This model lacks predictors and is an intercept-based model. We investigated whether the multilevel ordinal logistic regression model was more suitable than the single-level ordinal logistic regression model by using the Likelihood Ratio (LR) test. The LR test results showed a statistically significant outcome (p<0.05), suggesting that the multilevel ordinal logistic regression model was the best fit for single-level ordinal logistic regression analysis. Therefore, the LR test supports the use of a multilevel ordinal logistic regression model. We fitted four random effects models, and the final selection was based on the lowest deviance value (Table 3).

**Table 3:** Multilevel ordinal logistic regression analysis of factors associated with levels of anemia among 6–59 months of children in Ghana.

Variable	Null model	Model I (with level one variables)	Model II (with level II variables)	Model III (with level 1 and level 2 variables)
**Child age in months**				
6–23		2.5(2.2,2.9)		2.5 (2.2, 2.9)**
24–59		1		1
**Sex of child**				
Male		1.2 (1.1,1.4)		1.2 (1.1, 1.4)**
Female		1		1
**Birth order**				
1		1		1
2–3		1.3(1.0,1.6)		1.3 (1.1,1.6)*
4–5		1.4(1.0,1.8)		1.4 (1.1,1.9)*
≥6		1.6(1.2,2.2)		1.7(1.2,2.3)**
**Diarrhoea in the last two weeks**				
Yes		1.1(0.9,1.3)		1.1(0.9,1.3)
No		1		1
**Underweight status**				
Normal		1		1
Underweight		1.0 (0.8,1.3)		1.0 (0.8,1.2)
**Stunting status**				
Normal		1		1
Stunted		1.5(1.3,1.9)		1.5 (1.30, 1.89)**
**Maternal age**				
15–24		1.6(1.2,2.1)		1.67(1.2,2.1)**
25–34		1.2(1.0,1.5)		1.22(1.0, 1.4)*
35–49		1		1
**Maternal educational status**				
No education		1.1(0.8,1.6)		1.1 (0.8, 1.6)
Primary		1.2(0.8,1.7)		1.3 (0.9,1.9)
Secondary		0.9(0.6,1.2)		1.0 (0.7,1.3)
Higher		1		1
**Maternal anemia status**				
Not anaemic		1		1
Anaemic		1.4(1.3,1.7)		1.4(1.3,1.7)**
**Place of delivery**				
Home		1.1(0.9,1.4)		1.1 (0.9, 1.4)
Health facility		1		1
**Supplementation of iron during pregnancy**				
Yes		1		1
No		1.5(1.3,1.9)		1.0(0.9,1.2)
**Number of household members**				
≤5		1		
>5		1.0(0.8,1.2)		1.0(0.8,1.1)
**Household wealth status**				
Poorest		2.0(1.4,2.8)		1.6(1.1,2.4)*
Poorer		1.6(1.1,2.2)		1.4(1.0,2.0)*
Middle		1.8(1.3,2.4)		1.7(1.2,2.3)*
Richer		1.5(1.1,2.1)		1.5(1.1,2.0)*
Richest		1		1
**Availability of improved water source**				
Improved		1		1
Not improved		0.9(0.8,1.2)		1.0(0.8,1.3)
**Type of toilet**				
Improved		1		1
Unimproved		0.9(0.7,1.2)		1.0(0.8,1.2)
Open defecation		1.5(1.2,1.8)		1.1(0.9,1.4)
**Media exposure (N = 3533)**				
Yes		1		1
No		1.2(1.0,1.4)		1.2(1.0,1.4)*
**Drugs for Intestinal parasites**				
Yes		1		1
No		0.9(0.8,1.1)		0.9(0.8,1.0)
**Decision Autonomy**				
Low		1.0(0.9,1.2)		1.0(0.9,1.2)
Moderate		1.1(0.9,1.3)		1.0(0.9,1.3)
High		1		1
**Residence**				
Urban			1	1
Rural			1.4(1.2,1.6)	1.3(0.9,1.4)
**Regions**				
Western			1.3(0.8,2.2)	1.1(0.7,1.9)
Central			1.4(0.9,2.3)	1.0(0.6,1.7)
Greater accra			1	1
Volta			1.7(1.0,2.8)	1.3(0.8,2.1)
Eastern			1.0(0.6,1.6)	0.8(0.5,1.4)
Ashanti			1.0(0.6,1.6)	0.9(0.5,1.4)
Western North			1.1(0.7,1.9)	0.9(0.5,1.5)
Ahafo			0.8(0.5,1.3)	0.5(0.3,0.9)
Bono			1.0(0.6,1.7)	0.9(0.5,1.5)
Bono east			1.7(1.1,2.7)	1.1(0.7,1.8)
Oti			2.1(1.3,3.4)	1.3(0.8,2.1)
Northern			4.0(2.6,6.2)	2.3(1.4,3.7)**
Savannah			2.7(1.7,4.3)	1.7(0.9,2.7)
North East			3.5(2.2,5.4)	2.1(0.9,2.7)
Upper East			3.2(2.0,5.1)	2.4(1.4,3.9)**
Upper West			3.1(2.0,5.0)	2.4(1.5,3.9)**
**Random effect analysis results**				
LLR	-4039.3	-3735.1	-3947.5	-3694.5
Deviance (-2LLR)	8819.1	8496.5	8655.9	8404.7
AIC	8084.6	7534.3	7933.0	7485.1
BIC	8103.4	7734.3	8052.0	7785.0
LR-test	LR test vs. logit model: chibar2(01) = 33.1 Prob > = chibar2 <0.001			

** For level of significant, (eg; *P<0.05; **P<0.01; ***P<0.001; 1 = reference category)

AIC: Akaike Information Criteria, AOR: Adjusted Odds Ratio, BIC: Bayesian Information Criteria, LLR: Log-Likelihood Ratio, LR: Likelihood Ratio

### Proportional odds assumption

The test results showed that the proportional odds assumption was met (p = 0.26), and all variables in the model had a p-value greater than 0.05, indicating that the assumption was fulfilled for each variable individually.

### Factors associated with levels of childhood anemia

To discern the factors associated with anemia, a bi-variable analysis was carried out. The age of the child in months, the sex of the child, birth order (parity), the use of intestinal parasite drugs in the last six months, a history of diarrhoea in the past two weeks, being underweight, stunted growth, maternal age, maternal educational status, maternal anemia, the place of delivery, supplementation of iron during pregnancy, the number of household members, household wealth status, availability of an improved water source, type of toilet, media exposure, decision-making autonomy, residence, and region were all taken into account for the multivariate analysis (p<0.2). In the multivariable multilevel proportional odds model, the age, sex, birth order, stunting, maternal age, maternal anemia, household wealth status, media exposure, and region were all found to be significantly associated with the severity of anemia. The likelihood of experiencing higher levels of anemia among children aged 6–23 months was found to be 2.5 times [AOR = 2.5, 95% CI: 2.2, 2.9] more likely than among children aged 24–69 months. Male children had 1.26 times [AOR = 1.2, 95% CI: 1.1, 1.4] greater odds of developing anemia compared to female children. Children from birth orders of 2–3, 4–5, and ≥6 were respectively 1.3 times [AOR = 1.3, 95% CI: 1.1, 1.6], 1.4 times [AOR = 1.47, 95% CI: 1.14, 1.90], and 1.7 times [AOR = 1.7, 95% CI: 1.2, 2.3] more likely to have higher levels of anemia than children from single birth orders (parity). Furthermore, children who were stunted had 1.5 times [AOR = 1.5, 95% CI: 1.3, 1.8] higher odds of having higher levels of anemia. Additionally, children whose mothers were aged 15–24 years and 25–34 years were 1.67 times [AOR = 1.6, 95% CI: 1.2, 2.1] and 1.22 times [AOR = 1.2, 95% CI: 1.0, 1.4] more likely to have higher levels of anemia compared to children whose mothers were aged ≥35 years. Children with anaemic mothers were 1.4 times more likely [AOR = 1.4, 95% CI: 1.3, 1.7] to have a higher level of anemia than those with non-anaemic mothers. The odds of anemia in children from poorer households were 1.6 times [AOR = 1.6, 95% CI: 1.1 2.4] higher, while those in poorer, middle and richer households had 1.4 [AOR = 1.4, 95% CI: 1.0, 2.0], 1.7 [AOR = 1.7, 95% CI: 1.2, 2.3], and 1.5 [AOR = 1.5, 95% CI: 1.1, 2.0] times higher odds, respectively. Children from families with no media exposure had 1.2 times [AOR = 1.2, 95% CI: 1.0, 1.4] higher odds of higher levels of anemia compared to those with media exposure. The odds of anemia in children from Upper West, Upper East, and Northern regions were 2.43 [AOR = 2.4, 95% CI: 1.5, 3.9], 2.4 [AOR = 2.4, 95% CI: 1.4, 3.9], and 2.3 [AOR = 2.3, 95% CI: 1.4, 3.7] times higher than those in Greater Accra, according to (Table 3).

## Discussion

The prevalence of anemia in Ghana was found to be 49.1% [95% CI: 47.4%–50.7%], which suggests that childhood anemia continues to be a significant public health issue in the country. Despite the World Health Organization implementing combined measures to combat anemia, including iron supplementation and disease management (such as helminths infections and malaria), anemia remains a pressing healthcare concern in Ghana. However, this is lower than the prevalence reported in Liberia [30], South and Southeast Asia [31], India [32], Ethiopia [33], and Nigeria [34]. The potential reason may be that the implementation of anemia intervention and control programs is gradually impacting the population [35].

The final model revealed that several factors, including child age, sex, birth order, stunting, maternal age, anemia, household wealth, media exposure, and region, were significantly linked to increased odds of anemia severity. Additionally, we found that children aged 6–23 months were more likely to develop higher levels of anemia than those aged 24–59 months. This is in line with the studies conducted in India [32], Liberia [30], and Bangladesh [36]. A potential explanation for this phenomenon is that the reduction of iron reserves during pregnancy was most notable at six months of age. Additionally, the span between six and twenty-three months is a critical period for introducing complementary feeding and coming into contact with contaminated food and water, which can increase the probability of contracting intestinal infections such as typhoid, amoebiasis, giardiasis, ascariasis, and hookworm infections [37]. Therefore, targeted interventions, including timely introduction of iron-rich complementary foods, improved hygiene practices, and regular screening for intestinal infections, should focus on children aged 6–23 months to reduce their higher risk of anemia.

The association between male sex and increased odds of childhood anemia compared to female sex is consistent with studies conducted in Lao PDR [38], Bangladesh [36], and low-income countries [39]. One possible explanation for the higher risk of anemia in male children is their rapid growth and development during the first few years of life [37]. This increased demand for micronutrients such as vitamin A, folate, and iron, which are essential for their faster growth rates [15, 40, 41]. Specifically, during early childhood [42], boys require more iron to meet their increased demands. Programs addressing childhood anemia should include targeted nutritional strategies for male children, focusing on meeting their increased needs for iron and other essential micronutrients during early childhood.

Children born as multiple births are at a higher risk of anemia compared to those born as singleton births, consistent with reports from India [32] and Ethiopia [33]. This may be attributed to the fact that multiple births are often premature and have low birth weights, increasing their risk of malnutrition and anemia [43, 44]. Healthcare programs should prioritize nutritional support and close monitoring of children from multiple births, addressing their higher risk of malnutrition and anemia due to factors like prematurity and low birth weight.

In this study, stunted children had higher odds of developing higher levels of anemia than normal children. This is in line with studies reported in Ethiopia [33], Liberia [30], and South Asia [31] A potential reason could be an inadequate diet, which could result in a deficiency of vital nutrients required for the production of red blood cells and has been associated with decreased immunity and increased susceptibility to diseases and infestations. Furthermore, malnourished children are more prone to experiencing micronutrient deficiencies, such as iron, vitamin A, vitamin B12, and folic acid, which are essential for the synthesis of haemoglobin and DNA during the production of red blood cells, ultimately leading to anemia [38]. It is recommended that comprehensive nutrition interventions be implemented to address stunting, with a focus on ensuring adequate intake of essential micronutrients such as iron, vitamin A, vitamin B12, and folic acid, to mitigate the risk of anemia and promote optimal growth and development in children.

Children born to mothers aged 15–24 and 25–34 had a higher likelihood of experiencing higher levels of anemia compared to children born to their counterpart mothers, as reported in studies conducted in India [32]. This phenomenon could be explained by the lower dietary iron intake and increased demand for iron during menstruation, pregnancy, and lactation, which can have negative consequences [39]. It is essential to provide proper nutrition during the phase of rapid growth to achieve full growth potential, as inadequate nutrition may result in delayed and stunted growth in children and impaired organ remodelling. Young women are at risk of developing a range of health problems, including anemia, if they do not receive adequate nutrition, as they require additional micro- and macronutrients [40]. It is crucial that targeted maternal nutrition programs be implemented to ensure adequate micronutrient intake, particularly iron, during pregnancy and lactation, to reduce the risk of anemia in both mothers and their children, and support optimal growth and development.

Children born to anaemic mothers were found to have a higher likelihood of having higher levels of anemia than those born to non-anaemic mothers. This finding is consistent with the results of studies conducted in Ethiopia [33], South Asia [31], and Bangladesh [36]. One possible explanation for this is that both the mother and child may be exposed to similar infectious diseases, such as helminthiasis, malaria, and other infections that can interfere with red blood cell production and iron stores [41]. It is recommended that integrated maternal and child health interventions be implemented to prevent and manage maternal anemia, while simultaneously addressing the prevalence of infectious diseases such as helminthiasis and malaria, in order to reduce the risk of anemia in both mothers and their offspring.

Children from low-income households had a higher chance of having severe anemia compared to those from wealthy households, consistent with previous research in Nepal, Bangladesh, Nigeria, and Ethiopia [29, 33, 34, 36]. The reason for this disparity might be due to food insecurity, which can result in inadequate nutrition and a lack of essential nutrients, such as folate, iron, and vitamin B12 [42]. Furthermore, limited financial resources can lead to a lower likelihood of purchasing nutrient-rich foods and accessing healthcare services when needed. It will be better that policies and programs aimed at reducing childhood anemia prioritize improving food security and access to affordable, nutrient-rich foods for low-income households, while also enhancing access to healthcare services to address and prevent anemia in these vulnerable populations.

Similarly, children from households with no media exposure had a higher likelihood of experiencing severe anemia compared to those from households with media exposure, a finding that aligns with previous research from India [32] and Liberia [30]. This could be attributed to the media serving as a primary source of information, raising awareness about anemia causes and effective feeding practices to minimize its impact. It is suggested that initiatives be implemented to increase media-based health education in communities with limited media exposure, to raise awareness about anemia prevention, proper nutrition, and feeding practices, thereby reducing the incidence of severe anemia in children.

Children from the Northern, Upper East, and Upper West regions of Ghana were found to have significantly higher odds of having higher levels of anemia compared to those in the Greater Accra region. One possible reason for this disparity could be the high poverty rates and lower levels of education in these predominantly rural areas. Additionally, these regions have a high incidence of malaria transmission, which is a significant contributor to childhood anemia, as Plasmodium falciparum is a leading cause of the condition [44]. In contrast, the Greater Accra region had a lower prevalence of anemia in children, which is consistent with the existing literature [43]. It is advised that targeted interventions be introduced in the Northern, Upper East, and Upper West regions of Ghana, focusing on poverty alleviation, education improvement, and enhanced malaria control efforts, to address the high incidence of anemia in children in these areas.

This study had several strengths and limitations. The researchers employed a weighted pooled nationally representative recent DHS survey in Ghana and conducted a multilevel ordinal logistic regression analysis to obtain reliable estimates and standard errors. The sample size was sufficient to detect the true effects of the independent variables. However, it is essential to consider the limitations of the study, as it cannot establish a causal relationship between anemia and its predictors due to the use of cross-sectional data. Furthermore, the study was not able to explore all variables relevant to childhood anemia, as it relied on secondary data.

## Conclusions

Anemia is a critical problem for children aged 6–59 months in Ghana, and several factors have been identified as significant predictors of anemia severity, including age, sex, birth order, stunting status, maternal age, maternal anemia, household wealth status, media exposure, and region. To address this issue, it is recommended to improve access to media, target maternal anemia through interventions, strengthen family wealth, and support preconception care for mothers during pregnancy. Additionally, stunted children’s early detection and management should be enhanced to decrease childhood anemia.

## Abbreviations

ANC: Antenatal care
AOR: Adjusted Odds Ratio
CI: Confidence Interval
DHS: Demographic and Health Survey
EAs: Enumeration areas
GDHS: Ghana Demographic health survey
ICC: Intra-cluster Correlation Coefficient
KR: Kids Record
LLR: Log likelihood ratio
LR: Likelihood ratio
OR: Odds ratio
SDG: Sustainable Development Goal
WHA: World Health Assembly
WHO: World Health Organization
